# Aqueous based reflux method for green synthesis of nanostructures: Application in CZTS synthesis

**DOI:** 10.1016/j.mex.2015.12.003

**Published:** 2015-12-19

**Authors:** Sai Kiran Aditha, Aditya Dileep Kurdekar, L.A. Avinash Chunduri, Sandeep Patnaik, Venkataramaniah Kamisetti

**Affiliations:** Nanoscience Laboratory, Department of Physics, Sri Sathya Sai Institute of Higher Learning, Prasanthinilayam, 515 134 AP, India

**Keywords:** Reflux, CZTS, Green synthesis, Aqueous method, Nanostructures, Photovoltaics

## Abstract

The aqueous based reflux method useful for the green synthesis of nanostructures is described in detail. In this method, the parameters: the order of addition of precursors, the time of the reflux and the cooling rate should be optimized in order to obtain the desired phase and morphology of the nanostructures. The application of this method is discussed with reference to the synthesis of CZTS nanoparticles which have great potential as an absorber material in the photovoltaic devices. The highlights of this method are:

• Simple.
• Low cost.
• Aqueous based.

## Graphical abstract

**Figure:**
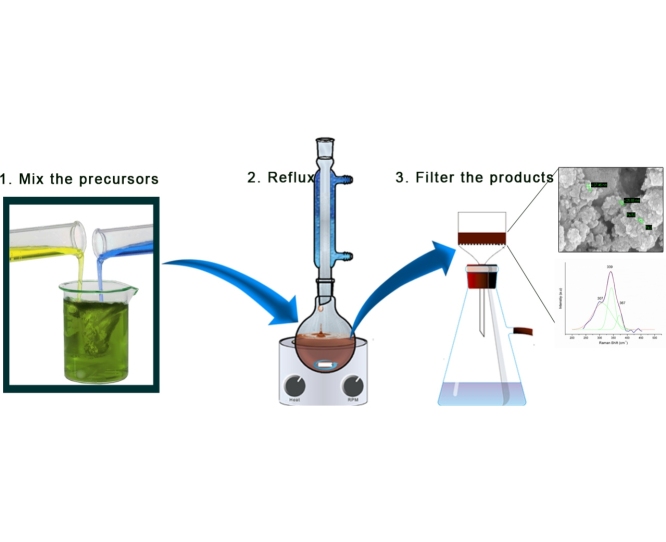

## Method overview

There exist many methods to prepare nanostructured materials with various sizes and shapes. These methods can be broadly categorized into vacuum based and non-vacuum routes. The vacuum based methods include sputtering [1], vacuum deposition [2], thermal evaporation [3], etc. The non-vacuum routes that are employed are electrodeposition [4], solution processing [5]. The vacuum based techniques are energy intensive requiring high vacuum and temperatures, thereby indirectly increase the effective cost. Most of these non-vacuum methods use toxic chemicals and organic solvents that are harmful to the environment. Therefore aqueous based methods that are easily scalable are highly desired.

The aqueous based reflux method is a simple, low cost method that gives the desired product with precise control over reaction parameters. This method has been used to synthesize various nanostructured materials such as nanoparticles [6], nanowires [7], nanorods [8], nanourchins [9], core–shell nanostructures [10], hierarchical nanostructures [11]. In this method the energy necessary for the reaction is supplied by heating the reaction solution over long periods of time. Through this method one can control the size, morphology and crystallinity of the materials by varying parameters such as the reaction time, concentration of precursors and the type of solvent employed [12], [13]. In this method, the parameters: the order of addition of precursors, the time of the reflux and the cooling rate should be optimized in order to obtain the desired phase and morphology of the nanostructures.

**The order of the addition of precursors**: In the case of reactions where multiple precursors are involved the order of reaction plays an important role. If the product is quaternary like CZTS then the chance of formation of secondary phases is very high. Therefore it is necessary to identify the various secondary phases that could form, find out the reactivity's and their formation energies. If one of the binary compounds is more stable than the others then such a reaction should be avoided. Thus the desired phase can be obtained by carefully choosing the order of the precursors.

**The time of the reflux**: After the initial nucleation, grain growth takes place and simultaneously the phase formation also takes place. If the reaction is stopped early, then there will be incomplete phase formation. On the other hand the increase in the time of reaction leads to a larger grain size and hence bulk crystals will be obtained. Therefore the time of reflux should be optimized in order to obtain a pure phase nanostructure.

**The cooling rate:** Many times this parameter is overlooked but it is an important factor. A reaction could be arrested by a rapid decrease in the temperature. At higher temperatures the kinetic energies of the molecules is high and the nucleation species are very mobile in the reaction system. As the reaction cools down, the mobility decreases and the product becomes stable. Slow cooling rate leads to defect free particles whereas rapid cooling of the solvated ions will end up in the formation of defective crystals.

CZTS (Copper Zinc Tin Sulfide), a quaternary chalcogenide, is an upcoming material with applications in photovoltaics as an absorber. It has an optimum bandgap of about 1.5 eV, and a high absorption coefficient of the order 10^4^ cm^−1^ [14]. The raw material costs of leading solar cell technologies are presented in Table 1. Clearly CZTS is an order of magnitude lesser in price compared to the other materials [15].

In this article we report the use of reflux method for the synthesis of CZTS nanoparticles.

The equilibrium equation of CZTS formation as reported in the literature [16] is

Cu_2_S + ZnS + SnS_2_ ↔ Cu_2_ZnSnS_4_

The formation of the binary sulphides is favourable at higher temperatures whereas at lower temperatures the equilibrium shifts towards the right hand side. Therefore the recrystallization of CZTS depends on the cooling rate.

At higher temperatures the Cu-rich phases are prone to form, leading to a greater number of defected particles. Tin sulphide is volatile and goes out of the system at temperatures above 450 °C [17].

Higher the temperature, more the defected particles and at temperatures above 550 °C, CZTS decomposes. Thus synthesis of CZTS at lower temperatures is preferable. Therefore, reflux method would be ideal as it can supply continuous energy for long time periods at a constant temperature.

## Method description

**Fig. 1:**
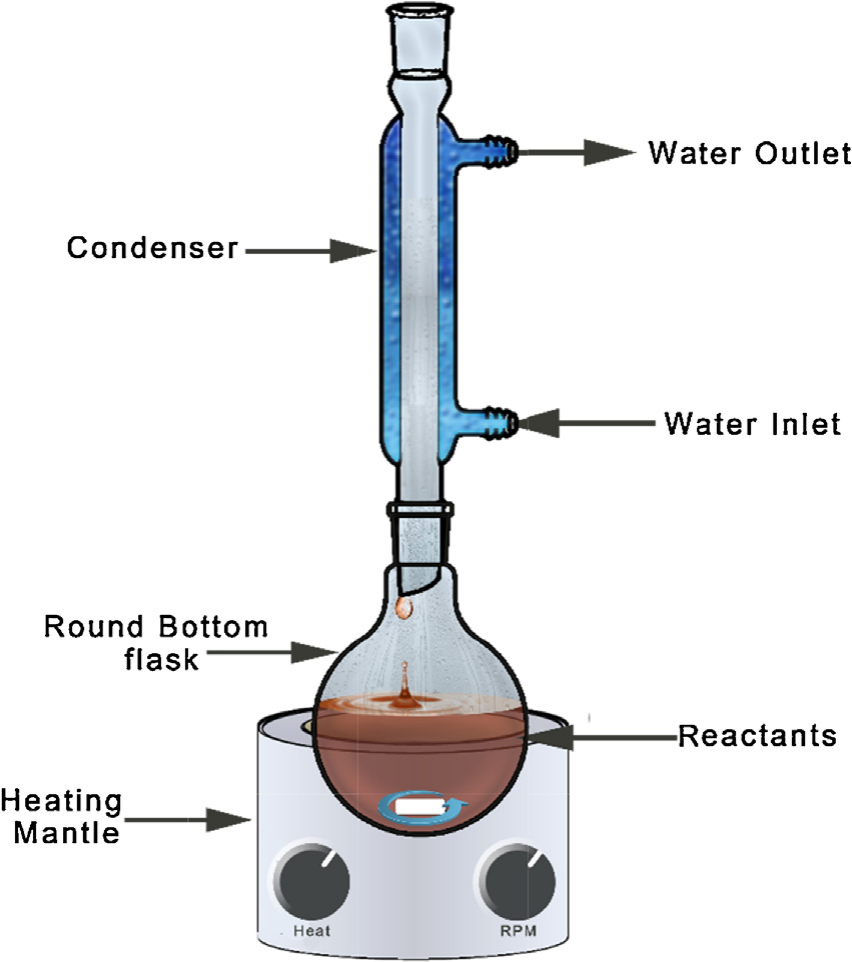
Schematic of reflux setup.

A typical reflux setup is shown in the schematic (Fig. 1). The reaction vessel (usually a round bottom flask) is fitted with a Liebig or Vigreux condenser which prevents the solvent vapours from escaping the system. The condenser has an outer jacket through which a coolant (usually chilled water) is circulated. The circulating water takes the temperature off the solvent vapours and in turn the vapours condense and fall back into the reaction vessel. Thus the total volume of the solution remains same and therefore can be heated over long time periods. In the reflux method the reaction temperature is the boiling point of the solvent used. Therefore, based on the need, the solvent can carefully be chosen to suit the reaction. If higher temperatures are needed an oil bath or a sand bath is employed. Often times a magnetic stirrer is used to stir the contents of the reaction vessel in order to uniformly distribute the heat.

The starting materials used are CuCl_2_, ZnCl_2_, SnCl_2_·2H_2_O and thio-urea. All the materials are pure and used without any further purification. Separate aqueous solutions of 1 molar Cupric Chloride, 0.5 molar zinc chloride, and 0.5 molar stannous chloride were prepared. Two molar aqueous solution of thio-urea is taken into the rbf and to it the tin chloride solution was added and stirred well till the solution turned clear. To it the Cu and Zn solutions were added simultaneously. The solution was refluxed for 8 h and was allowed to gradually cool to room temperature. Initially the solution was milky white and upon heating turned greyish-black. The products were filtered, washed with distilled water and ethanol several times. Then the obtained powder was dried under vacuum.

The reaction mechanism:

$$\begin{array}{c} 2{\text{CuCl}}_{2}+{\text{ZnCl}}_{2}+{\text{SnCl}}_{2}\cdot 2{\text{H}}_{2}\text{O}+4\text{SC}{\left({\text{NH}}_{2}\right)}_{2}+8{\text{H}}_{2}\text{O}\to \\ \left\{{\text{Cu}}^{2+}\to {\text{Cu}}^{+},{\text{ Sn}}^{2+}\to {\text{Sn}}^{4+}\right\}\\ 2{\text{Cu}}^{2+}+{\text{Zn}}^{2+}+{\text{Sn}}^{2+}+4{\text{ S}}^{2-}\to {\text{Cu}}_{2}{\text{ZnSnS}}_{4}+{\text{CO}}_{2}+8{\text{ NH}}_{4}\text{Cl}. \end{array}$$

## Validation of the method

In order to confirm the formation of CZTS and in turn validate the method, characterization of the material was carried out. Phase of the material is established by X-ray diffraction analysis and Raman spectroscopy. The bandgap of the material is determined from the UV–Vis absorption studies. The size and the morphology of the synthesized sample is seen from the scanning electron micrograph. The elemental composition is given by the EDX analysis.

**Fig. 2:**
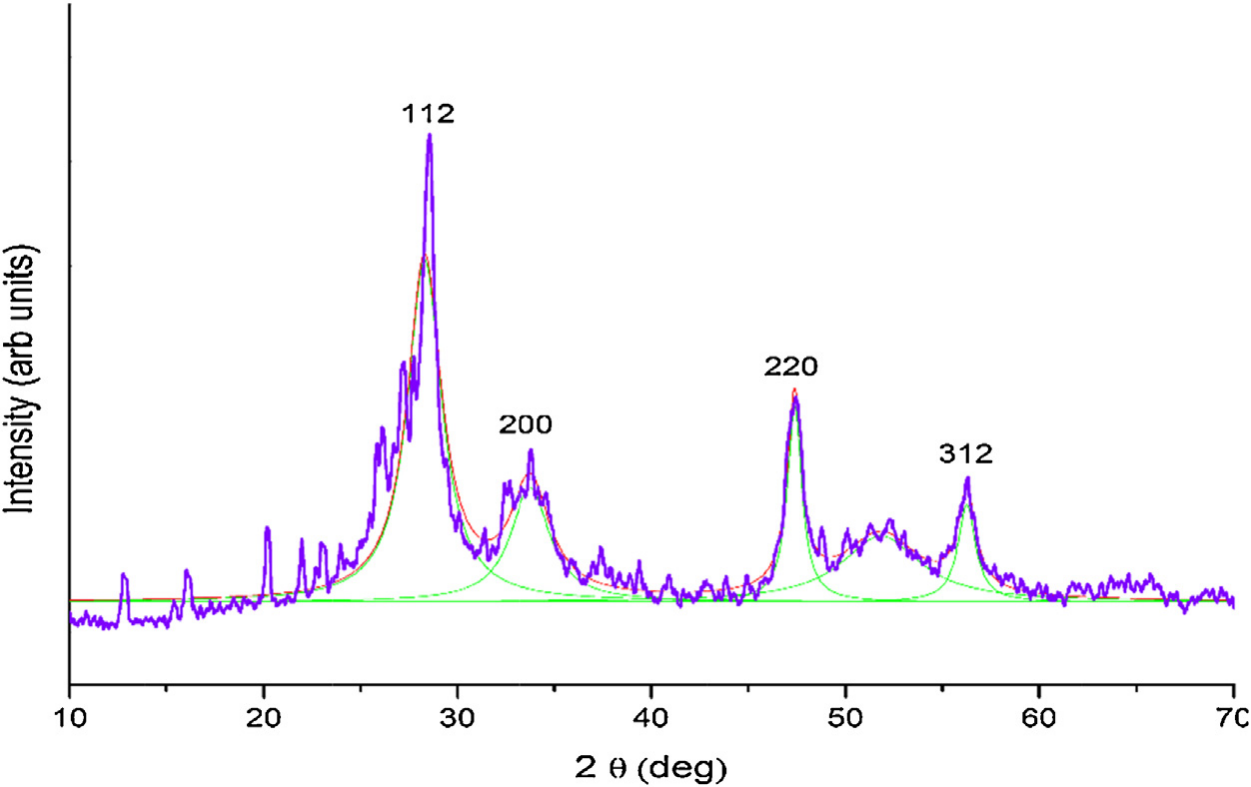
XRD pattern of the as synthesized CZTS sample.

**Phase Identification**: Phase identification was done by using a Panalytical X-ray diffractometer with Cu Kα radiation *λ* = 1.5406 A°, step size = 2°/min. The XRD pattern of the as synthesized powder is shown in Fig. 2. The peaks at 2 theta 28.54° 33.76°, 47.5° and 56.24° correspond to the (112), (200), (220) and (312) planes of the kesterite CZTS (JCPDS card no #26-0575) which is in tune with the literature [18].

From the XRD data the average crystallite size is calculated using the Scherrer Formula:

$$D=\frac{0.9\lambda }{\beta \cos\theta }$$

where *D* is the average crystallite size (diameter), *λ* is the wavelength of the incident radiation, *θ* is the Bragg angle and *β* is the full width (in radians) of the peak at half the maximum intensity.

The average crystallite size calculated from the above formula is 2.5 nm. Since the XRD patterns of CZTS and ZnS are similar, the formation of the phase needed further confirmation, which was achieved by the Raman spectroscopy.

**Fig. 3:**
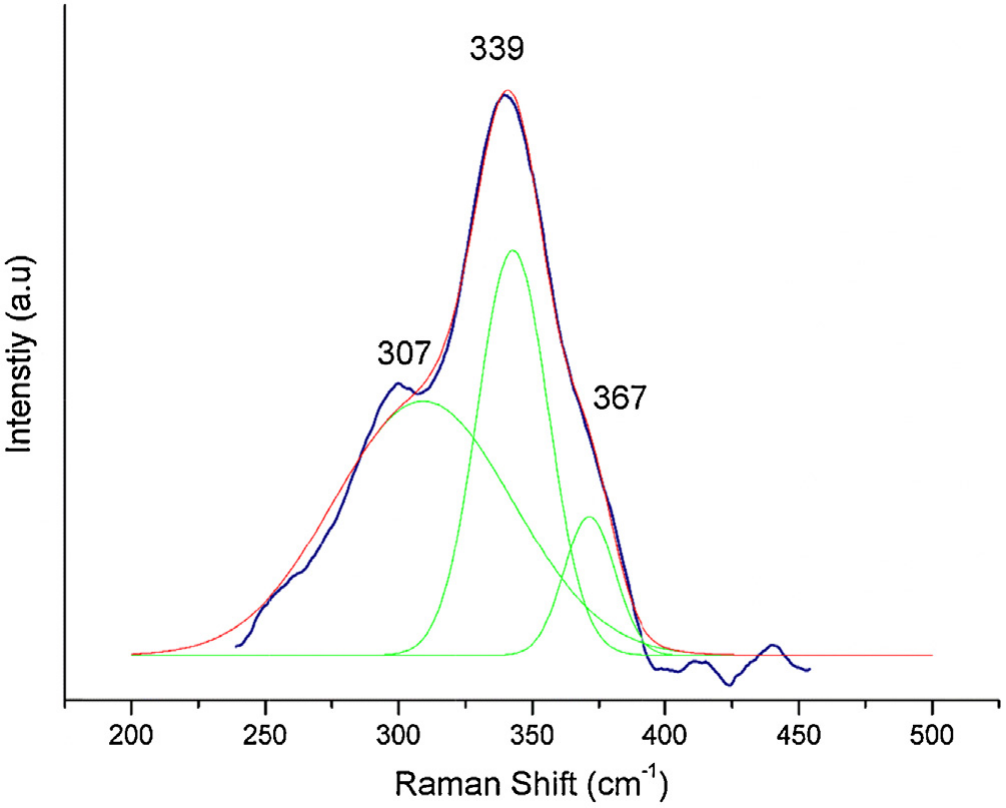
Raman spectrum of the as synthesized sample.

For the Raman spectra, a Horiba iHR 550 Raman spectrophotometer illuminated with a 532 nm laser beam was used. The peaks at 307 cm^−1^ and 339 cm^−1^ respectively, correspond to the CZTS phase [19]. Fig. 3 shows the Raman spectrum of the as synthesized sample.

## Band gap calculations

**Fig. 4:**
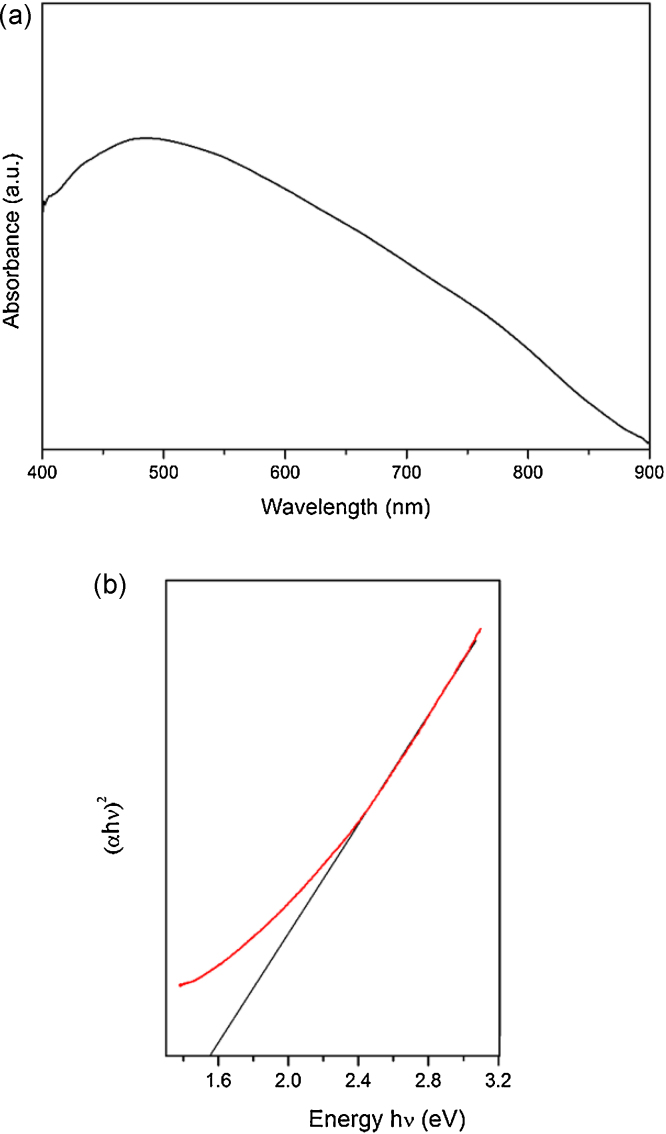
(a) UV–Vis spectrum of the as synthesized sample. (b) Tauc plot showing the band gap.

The optical band gap studies were performed with a Shimadzu UV 2450 uv–vis spectrophotometer. The absorbance spectrum was recorded and Fig. 4a shows the UV–Vis absorbance spectrum. The bandgap value is obtained from the tauc plot where (*αhν*)^2^ is plotted as a function of incident photon energy; *hν*. The linear part of the curve is extrapolated and the intercept is the bandgap of the material. Fig. 4b shows the Tauc plot. Upon extrapolation, the band gap is found to be 1.56 eV, which is consistent with the literature [20].

## FESEM and EDX

**Fig. 5:**
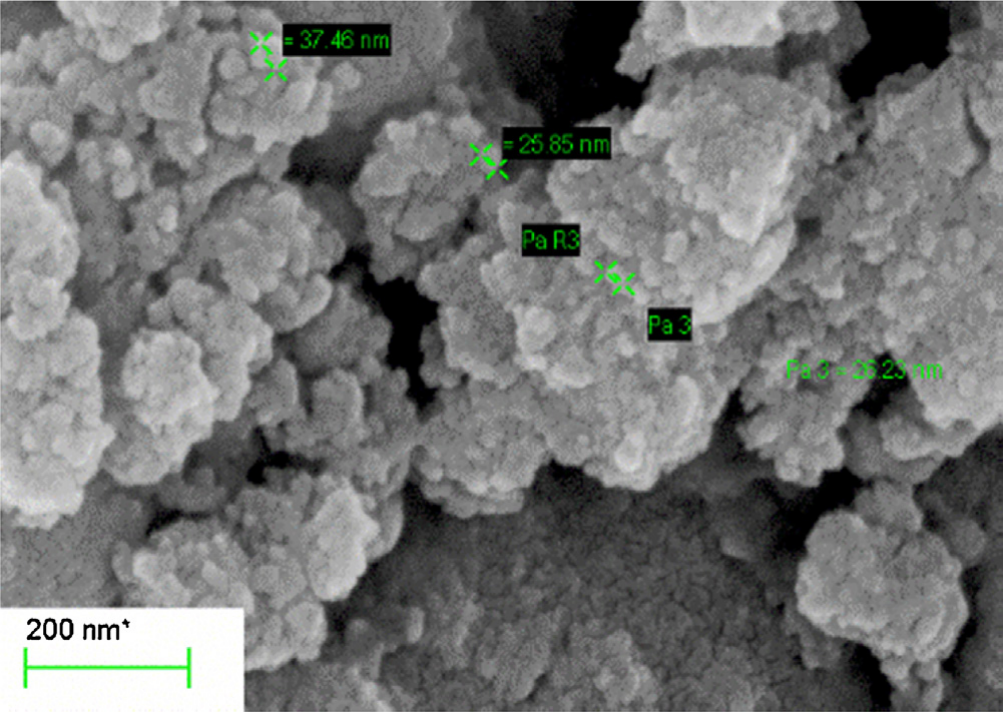
SEM of the as synthesized CZTS sample.

**Fig. 6:**
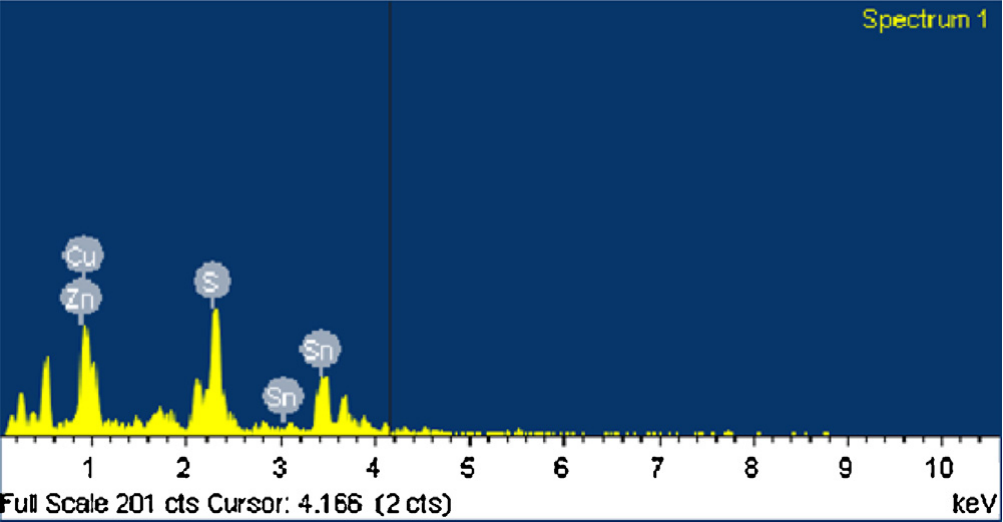
EDX pattern of the as synthesized CZTS sample.

The electron micrographs were taken using a ZEISS scanning electron microscope fitted with an Energy dispersive X-ray detector. The elemental ratios as obtained from the EDX detector were presented in Table 2. The corresponding electron micrograph is shown in Fig. 5. The particle size analysis from the SEM micrograph is done by using ImageJ software. From the SEM image, the morphology is known to be spherical with an average particle size of 29 nm. The EDX results indicate that the elements in the sample are stoichiometric indicating a pure phase (Fig. 6).

## Figures and Tables

**Table 1:** Table showing the raw material cost per watt of leading solar cell technologies.

S. no.	Compound	Raw material cost (/*W*)
1.	CZTS	4.9E−3
2.	CdSe	1.2E−2
3.	CIGS	2.3E−2
4.	CdTe	9.7E−2
5.	InP	2.3E−1
6.	GaAs	2.5E−1

**Table 2:** Elemental ratios obtained from the EDX results.

Element	Atomic%	Weight %
S K	54.96	33.12
Cu L	21.67	25.93
Zn L	11.25	13.93
Sn L	12.12	27.02

Totals	100.00	100.00
